# Inhibitory control may not explain the link between approximation and math abilities in kindergarteners from middle class families

**DOI:** 10.3389/fpsyg.2015.00685

**Published:** 2015-05-22

**Authors:** Leanne Keller, Melissa Libertus

**Affiliations:** ^1^Department of Psychology, University of Pittsburgh, Pittsburgh, PA, USA; ^2^Learning Research and Development Center, University of Pittsburgh, Pittsburgh, PA, USA

**Keywords:** approximate number system, math, inhibitory control, numerical estimation, early childhood

## Abstract

Past research suggests that individual differences in the acuity of the approximate number system (ANS) are associated with children’s math abilities. However, some recent work has argued that these associations can be explained through shared reliance on inhibitory control. Here, we test this claim in two separate experiments. In Experiment 1, forty-two 5- and 6-year-old children completed a non-symbolic number comparison task to assess ANS acuity as well as standardized experimenter-administered assessments for inhibitory control and math ability. Children’s accuracy in the number comparison task and scores on the math assessment were significantly correlated, even when controlling for performance on the inhibitory control task. To rule out that our findings were due to the nature of the inhibitory control task, in Experiment 2, we administered a different, computerized inhibitory control task, and similar tasks to assess ANS acuity and math ability as in Experiment 1 to children aged 3–6 years (*N* = 169). Similar to the result of Experiment 1, we found that associations between accuracy in the number comparison task and math ability persisted when controlling for performance on the inhibitory control task. Together these results suggest that ANS acuity is uniquely associated with early math abilities, independent of the effect of inhibitory control at least in children from middle- to high-SES families.

## Introduction

Several large longitudinal studies suggest that children’s early math abilities are predictive of later academic success and socio-economic status in adulthood (Duncan et al., 2007; Ritchie and Bates, 2013). Given the importance of early math abilities, research has aimed to identify factors that promote children’s math abilities. In addition to important contextual factors in the home and the classroom (e.g., Klibanoff et al., 2006; Melhuish et al., 2008), children’s own cognitive abilities, including domain-general and domain-specific ones, appear to predict their math abilities. In this paper, we will examine the interplay between one domain-general cognitive ability, namely inhibitory control, and one domain-specific cognitive ability, namely the approximate number system (ANS), in their role for early math abilities.

Although characterizations vary across the literature, executive functions are typically defined as a subset of cognitive skills that includes working memory, cognitive flexibility, and inhibitory control, all of which are needed to successfully solve problems (e.g., Zelazo and Müller, 2002; Jurado and Rosselli, 2007; Garon et al., 2008; Liew, 2012). These executive functions are seen fairly early in life and develop rapidly during the preschool and kindergarten years (see Zelazo and Müller, 2002; Carlson, 2003). Past research has demonstrated that this set of domain-general cognitive skills promotes children’s early academic skills and that these associations appear to persist throughout the later elementary school years (McClelland et al., 2006; St Clair-Thompson and Gathercole, 2006). Several researchers have suggested that associations between executive functions and math are not unique and that these cognitive skills simply promote academic achievement across domains (St Clair-Thompson and Gathercole, 2006; Bull et al., 2008). However, some recent evidence suggests that executive functions may be particularly predictive of children’s math achievement as opposed to reading or academic achievement more broadly. In a longitudinal study addressing children’s cognitive development across the transition to formal schooling, executive functions at age 4 years, measured as a unitary construct, significantly predicted later math ability when holding reading skills and general cognitive abilities constant (Clark et al., 2010). Thus, executive functions may be particularly important for children’s developing math skills, over and above larger relations to broader domains of cognitive abilities.

Although several studies have addressed the effect of executive functions in combination with one another, separate skills in this subset may uniquely predict children’s academic achievement. For instance, inhibitory control predicts children’s academic achievement across domains in early childhood, controlling for general intelligence as well as other components of executive function (Blair and Razza, 2007). Additionally, there is some evidence that these links may be causal, as growth in behavioral regulation predicts children’s growth in literacy, math, and vocabulary skills across the preschool years (McClelland et al., 2007). Thus, inhibitory control appears to be an important skill underlying early academic achievement.

Some evidence has highlighted the importance of inhibitory control as especially critical for math learning. Recent neurological work supports this argument, claiming that inhibitory control and math activate similar brain regions during childhood (Houdé et al., 2010). Behavioral research appears to echo this finding; out of the three components of executive function, only inhibitory control uniquely contributed to children’s math scores in preschool when controlling for other executive functions and vocabulary (Espy et al., 2004), suggesting that inhibitory control may be particularly important during the transition to formal schooling. Though some research with older children has found unique effects on math abilities for multiple facets of executive function, inhibitory control remains a significant predictor of children’s math, even when accounting for working memory and cognitive flexibility (Bull and Scerif, 2001). In a recent meta-analysis of studies assessing associations between inhibitory control and both reading and math skills in preschoolers and kindergarteners, effects of inhibitory control were significantly stronger on math compared to reading (Allan et al., 2014). Further, larger effects of inhibitory control on math abilities were found in studies that used behavioral measures of inhibitory control as well as those that measured inhibitory control with more decontextualized and non-affective tasks (i.e., “cool” inhibitory control as opposed to “hot” inhibitory control, see Zelazo and Müller, 2002; Willoughby et al., 2011, for more on this distinction). Finally, inhibitory control deficits appear to be present among children with developmental dyscalculia, a learning disability specific to deficits in math ability (Szucs et al., 2013). In sum, all of these findings suggest that domain-general cognitive skills, especially inhibitory control, foster children’s early math learning above and beyond the effects of other executive functions and cognitive skills and the effects on more general academic abilities.

### Influence of Math-Specific Cognitive Abilities: The Approximate Number System

In addition to the established associations between children’s domain-general cognitive skills such as inhibitory control and math ability, some evidence suggests that number-specific cognitive abilities also play a role for math ability. One such component that has been identified is the ANS, a rudimentary cognitive system that represents and allows for the manipulation of numerical estimates of objects in the environment (e.g., the number of apples on a tree or number of voices in a crowded room) that has been observed in humans and several non-human animal species (for review, see Libertus and Brannon, 2009; Brannon et al., 2010). The precision of these estimates represented by the ANS varies as a function of overall quantity, i.e., the precision decreases with increasing quantity. Thus, comparisons of ANS representations are constrained by Weber’s Law, i.e., the ratio between the numbers to be compared determines discriminability (Dehaene, 1992).

Developmental research suggests that the ANS is present at birth (Izard et al., 2009), though representations are much less precise than those of older children and adults. Newborns have been shown to discriminate numerosities that differ by a ratio of 1:3 (e.g., 4 and 12) while 6-month-olds have been shown to discriminate between numerosities that differ by a ratio of 1:2 (e.g., 8 and 16; Xu and Spelke, 2000; Lipton and Spelke, 2003). Throughout development, the ANS slowly becomes more refined, as children between 3 and 6 years of age typically progress from discriminating between ratios of approximately 2:3 to ratios of quantities closer to 6:7 (Halberda and Feigenson, 2008).

Although the ANS is clearly distinct from the formal symbolic system needed to represent numerical information for exact calculation and complex mathematics that children start to acquire in early childhood, there is a wealth of empirical evidence to suggest that the two may be linked (Feigenson et al., 2013; Chen and Li, 2014; Fazio et al., 2014). Surprisingly large individual differences in ANS acuity exist and are related to performance on standardized math assessments during adolescence and adulthood (Halberda et al., 2008; DeWind and Brannon, 2012; Libertus et al., 2012; Lourenco et al., 2012), even when controlling for broader cognitive abilities such as verbal ability or working memory. Importantly, these associations are also seen in preschoolers and kindergarteners and thus appear prior to the start of formal math education. Children’s success at adding approximate, non-symbolic representations of quantities predicts performance on standardized math assessments at 5 years of age, suggesting an underlying connection between the ANS and math in early childhood (Gilmore et al., 2010). Furthermore, this association appears to hold even when assessing acuity of the ANS in isolation from mathematical operations like addition, as well as when controlling for children’s age and vocabulary development (Libertus et al., 2011). Longitudinal research has also demonstrated that ANS acuity in infancy and early childhood predicts later math ability (Mazzocco et al., 2011; Libertus et al., 2013; Starr et al., 2013). This association between acuity of the ANS and math may not be completely linear and may vary across individuals, as correlations appear to be stronger for children with lower math scores (Bonny and Lourenco, 2013). Similarly, children with developmental dyscalculia have significant deficits on non-symbolic number comparison tasks assessing ANS acuity (e.g., Landerl et al., 2004; Piazza et al., 2010; Mazzocco et al., 2011). Although the directionality and potential mechanisms to explain this association have yet to be uncovered (see Feigenson et al., 2013), prior research suggests that the ANS and math ability are connected, especially in early childhood prior to formal math instruction.

### Can Inhibitory Control Explain the Link Between ANS Acuity and Math?

Despite this evidence for an association between math ability and ANS acuity, two recent studies have called into question the nature of this relation. Among a sample of low-income children enrolled in Head Start, Fuhs and McNeil (2013) found marginal effects of ANS acuity on math ability that, when further probed, appeared to exist only for trials of the ANS acuity task that required children to ignore irrelevant perceptual features of the stimuli (i.e., trials in which the side with more dots had less overall surface area). Importantly, this association was reduced to non-significance when accounting for children’s inhibitory control. The authors argue that trials in which numerosity and surface area were negatively correlated required increased inhibitory control to ignore the misleading information presented by the stimuli in terms of surface area and to focus exclusively on number, and thus correlations between accuracy on these trials and math were fully explained by the components of inhibitory control tapped in these trials (Fuhs and McNeil, 2013). Although these findings may be attributable to the limited exposure to math concepts prior to schooling that the children in this sample experienced, they do call into question the general claim that children’s math ability is supported by underlying number-specific cognitive abilities.

Some additional recent work with more heterogeneous populations appears to replicate these effects. Gilmore et al. (2013) found similar patterns of associations between math ability and success on certain trial types in ANS accuracy tasks: performance on trials where overall surface area and numerosity were negatively correlated (i.e., more inhibitory control was needed) was associated with math ability, whereas performance on trials where surface area and numerosity were positively correlated was unrelated to math ability. Additionally, although performance across all trials significantly predicted children’s math ability, this effect dropped to non-significance when accounting for variation in inhibitory control (Gilmore et al., 2013). These findings suggest that observed correlations between ANS acuity and math ability may reflect the shared involvement of inhibitory control in both domains.

Despite this existing work, it is unclear how inhibitory control may relate to children’s math abilities as well as acuity of the ANS. Particularly, there is some debate regarding the nature of inhibitory control as a construct. Although several studies with adults and children have found that individual measures of inhibitory control load onto single latent factors of inhibitory control in confirmatory factor analyses (Miyake et al., 2000; Lehto et al., 2003), there is also some evidence that different types of inhibitory control tasks demonstrated divergent developmental trajectories, suggesting there may be important underlying subdomains of inhibition (Huizinga et al., 2006). In particular, Nigg (2000) has identified three overarching domains of inhibitory control: executive inhibition, motivational inhibition, and automatic attention inhibition. Two subdomains of executive inhibition, interference control and behavioral inhibition, are often used as indicators of inhibitory control as we refer to it here. Interference control is called upon in tasks presenting multiple sources of conflicting information, such as the Stroop task (e.g., Gerstadt et al., 1994) or Flanker task (e.g., Eriksen and Eriksen, 1974). Behavioral inhibition, on the other hand, requires the suppression of a proponent response and is measured through tasks such as the go/no-go task (e.g., Rubia et al., 2001). Children’s performance on these two types of tasks appears to be correlated though not identical, suggesting that these domains are linked yet distinct from one another (Archibald and Kerns, 1999). Additional distinctions in inhibitory control tasks have been made between simple and complex inhibitory control, where complex inhibitory control tasks also require working memory, as well as between whether the tasks require the inhibition of a reinforcing or automatic response or of a verbal or motor response (Archibald and Kerns, 1999; Garon et al., 2008). Further work establishing how these varying domains of inhibitory control operate in the associations between ANS acuity and math ability is warranted.

### The Current Study

Given this existing research suggesting that associations between ANS acuity and math ability may be attributable to aspects of inhibitory control, in the present study we sought to replicate and further examine these associations in two samples of children across a wider age range and using different measures of inhibitory control. Specifically, in Experiment 1 we assessed whether associations between ANS acuity and children’s math ability persisted when controlling for inhibitory control among a sample of 5- and 6-year-old children. Here, we used an experimenter-administered standardized test of inhibitory control that is part of the Developmental NEuroPSYchological Assessment-Second Edition (NEPSY-II; Korkman et al., 2007), which was used by Gilmore et al. (2013) in their study and that may tap into the interference control domain of inhibitory control. In Experiment 2, we expanded this age range and included a larger sample in order to test whether ANS acuity and math ability were related when controlling for inhibition as well as when focusing on specific conditions in the ANS task and on specific age ranges. Moreover, we included a computerized task to measure inhibitory control (Conners’ Kiddie Continuous Performance Test; K-CPT; Conners, 2006) that more closely aligned with behavioral inhibition in order to examine whether the specific task may explain differences in previous results.

## Experiment 1

### Methods

#### Participants

Participants included forty-two 5- to 6-year-old children (*M* = 5 years 11.93 months, SD = 7.28 months), 19 of whom were female. Thirty-six children (86%) were identified as Caucasian, two as African–American (5%), one as Asian (2%), two as multi-racial (5%) and one as other (2%). Additionally, in 95% of the families, either the child’s mother or father had a college degree. Parents of all children provided informed written consent prior to their child’s participation, and all children received a prize (e.g., stuffed animal, book, lunch box), for their participation.

#### Procedure

Children completed all tasks in a single session in the laboratory. The University of Pittsburgh’s Institutional Review Board approved this study. Generally, a trained experimenter first administered the number comparison task as a measure of ANS acuity, followed by standardized tests for mathematical ability, and inhibitory control.

### Measures

#### Number Comparison Task

**FIGURE 1:**
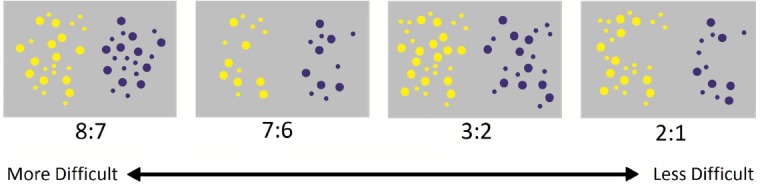
**Sample stimuli used in the non-symbolic number comparison task in Experiment 1**.

To assess the acuity of the ANS, children completed a non-symbolic number comparison task similar to that used by Halberda et al. (2008). Children saw sets of yellow and blue dots and, for each display, were asked to report which color was more numerous. In each image, yellow dots appeared on the left half of the screen and blue dots on the right and in half of the trials the yellow dots were more numerous and in the other half, the blue dots were more numerous. In one third of all trials (Correlated Trials), the average dot size was held constant between the two colors, such that the more numerous dots also had more overall surface area. In an additional third of all trials (Equated Trials), the total surface area was held constant across the blue and yellow dots. In the final third of trials (Anti-correlated Trials), the total perimeter was held constant, i.e., the dots on the more numerous side of the display took up less overall surface area than the dots on the less numerous side. Inhibitory control may be called upon more heavily in Anti-correlated and, to a lesser extent, Equated trials, given that children would need to ignore the irrelevant or even missing leading information regarding differences in surface area. This general pattern of associations has been found in past research utilizing these same conditions (Fuhs and McNeil, 2013). For all trials, dot size was on average 36 pixels in diameter and varied within sets (allowed variation = 20%). Stimuli were displayed for 1,500 ms on a 23′ computer monitor followed by a blank screen until the participants responded. The number of dots in each set ranged from 12 to 36. Example stimuli are shown in Figure 1.

The researcher first explained the task to each child and gave him/her six practice trials (two 1:3-ratio trials, followed by one from each of the four ratios used in test, i.e., 1:2, 2:3, 6:7, and 7:8) with pre-selected stimuli presented in a random order. Children were instructed to say “yellow” or “blue” as soon as they knew the answer and to attempt to respond accurately for as many trials as possible. The experimenter who was seated next to the child but was unable to see the computer screen entered the answer by pressing one of two keys on the keyboard as soon as the child responded. This method of collecting children’s responses has been used successfully in past work with children in this age range as it eliminates the need for children to learn which button to press for a given response allowing them to focus on the task itself (Libertus et al., 2011, 2013). For the first few practice trials, the researcher prompted the child with questions such as “Do you think there are more yellow dots or more blue dots?” and provided verbal feedback. Children who did not appear to understand the instructions or performed poorly on these practice trials repeated these six trials an additional time. Children then received a total of 72 test trials, i.e., 18 trials for each of the following ratios between the less and more numerous numerosities: 1:2, 2:3, 6:7, and 7:8. For test trials, the researcher made no prompts and gave no feedback about the correctness of the answer.

#### Mathematical Ability

We administered Form A of the Test of Early Mathematics Ability (TEMA-3; Ginsburg and Baroody, 2003). The TEMA-3 measures numbering skills (e.g., verbally counting the number of objects on a page), number-comparison facility (e.g., determining which of two spoken number words is larger), numeral literacy (e.g., reading Arabic numerals), mastery of number facts (e.g., retrieving multiplication facts), calculation skills (e.g., solving mental and written addition and subtraction problems), and number concepts (e.g., answering how many tens are in one hundred). The TEMA-3 has been normed for children between the ages of 3 years 0 month and 8 years 11 months.

#### Inhibitory Control Task

To assess children’s inhibitory control, more specifically their interference control ability, children completed the Inhibition subtest of the NEPSY-II (Korkman et al., 2007), a neuropsychological battery of tests for children aged 3 through 16. The NEPSY-II includes assessments of multiple domains of cognitive and sensorimotor development and the reliability and validity of the NEPSY-II subtests have been established in previous work (Davis and Matthews, 2010).

For 5- to 6-year-old children, the inhibition subtest consists of two stages: naming and inhibition. Children completed these stages for two sets of stimuli: shapes, which were squares or circles, and arrows, which pointed either up or down. All children completed both stages for shapes first, followed by arrows. During the naming stage, the researcher introduced the task and directed the child to label the shapes or arrows by saying “circle”/“square” or “up”/“down”, respectively. The researcher demonstrated this rule with a set of eight shapes or arrows and pointed to each image as it was labeled. The child was then asked to label the same practice set. If the child made fewer than five mistakes on these eight practice images, they then proceeded to the test phase. Children were allowed to correct any mistakes; self-corrected mistakes were not counted toward the five mistakes during this practice. For shapes and for arrows, all children advanced to the naming test phases.

For the test phase, children were presented with 40 images (either squares and circles or up and down arrows) arranged in five rows of eight. Children were instructed to name each image on the page as quickly as they could. During the test phase, the researcher recorded the time from when the child labeled the first shape on the page to the last as well as the number of uncorrected and self-corrected mistakes made by the child. Skipped images were counted as uncorrected mistakes. Several children did not read the stimuli in the correct order (i.e., skipped rows of images or read down the page instead of across; *n* = 9) in these cases, the experimenter attempted to redirect the child and instructed the child to point to the stimuli as he or she named each to ensure accurate scoring. For several children, this included restarting the naming test trials (*n* = 2), asking the child to name a missed row at the end of the naming test trial (*n* = 4), omitting the time spent on a repeated row (*n* = 2), or correcting the child at the end but making no adjustment (in the case of the child who read down the page instead of across; *n* = 1). When children skipped or repeated rows, the researcher kept time such that the time recorded represented the amount of time needed for the child to name each shape or arrow once, though not in the correct order.

After naming the shapes or arrows, children then progressed to the inhibition stage. The researcher reintroduced the set of eight images used as practice during the naming stage and instructed children to name the opposite response for each image (e.g., to say “circle” for each square shape or say “up” for each down arrow). Just like in the naming stage, the researcher demonstrated this rule with the eight practice stimuli, repeated the rule, and instructed children to label the practice set. If the child made fewer than five mistakes, he or she then completed the test trials. For shapes and for arrows, two children and one child did not advance to the inhibition test phases, respectively. Test stimuli, scoring, and timing in the inhibition stages were identical to those of the naming stage. Again, several children did not complete the task in the correct order and had to restart (*n* = 1) or repeat a missed row at the end (*n* = 2). Data from these children were included in our analyses as long as the scoring and timing were unaffected and representative of the child’s performance had he or she responded to each item once.

### Results

#### Data Analyses and Descriptive Statistics

Children’s performance on the number comparison task was quantified as the percentage of correct responses. Children’s accuracy across the three surface area trial types did not differ significantly from one another, *F*(2,90) = 1.99, *p* > 0.05; hence, we collapsed performance across trial types for all further analyses. Overall accuracy ranged between 41.67 and 86.11%, with an average of 68.12% (SD = 11.73%). Children’s raw scores on the TEMA-3 were converted to standard math ability scores, which ranged from 77 to 145 with an average score of 112.57 (SD = 16.64). Children’s performance on the naming and inhibition stages of the NEPSY-II inhibition subtest was converted to scaled scores based on the number of errors made and the completion time for each stage across the two sets of stimuli. Scaled scores for the two stages were then compared to create a contrast score, with higher scores representing better inhibitory control (i.e., more congruent scaled scores between the naming and inhibition stages). Three children were unable to complete the test phase of the inhibition stage for one or both sets of stimuli due to inadequate performance on the practice set; these children were removed from further analyses. Of the remaining 39 children who completed the NEPSY-II inhibition subtest, scores ranged from 4 to 15 with a mean of 9.92 (SD = 2.66).

#### Relation Between ANS Acuity and Mathematical Ability

To examine associations between ANS acuity and mathematical ability, we correlated children’s performance on the number comparison task with their standardized math scores on the TEMA-3. Children’s math ability scores were significantly correlated with ANS accuracy, *r*(40) = 0.42, *p* = 0.01, replicating previous findings (Inglis et al., 2011; Libertus et al., 2011, 2013; Bonny and Lourenco, 2013). Further, children’s contrast scores on the NEPSY-II inhibition subtest were positively correlated with standardized math ability scores, *r*(37) = 0.37, *p* = 0.02, but not with ANS accuracy, *r*(37) = 0.23, *p* = 0.17, suggesting that our measure of ANS acuity does not merely tap into inhibition skills.

Nevertheless, to directly assess whether the link between ANS acuity and math ability was not simply a reflection of associations with inhibitory control, we conducted partial correlations between ANS acuity and math ability controlling for inhibition. Children’s ANS accuracy and math ability scores were positively and significantly associated, even when controlling for inhibition contrast scores and child age, *r*(35) = 0.36, *p* = 0.03.

### Discussion

The results of Experiment 1 demonstrate that, consistent with past research, ANS acuity is significantly and positively associated with children’s math ability. Furthermore, these associations remained significant when holding inhibitory control constant suggesting that these math-specific cognitive abilities serve as a unique predictor of mathematics. These findings conflict with past research that argues that effects of ANS acuity on math can be fully explained by inhibitory control (Fuhs and McNeil, 2013; Gilmore et al., 2013). Unlike previous studies, we did not find any significant correlations between inhibitory control and ANS acuity. One possibility for these discrepancies could be subtle task differences. Hence, we conducted a second experiment in which we used the same computerized task to assess ANS acuity as in Experiment 1 but also used a computerized task to assess inhibitory control that tapped into children’s behavioral inhibition as opposed to interference control. Moreover, we tested a new, larger sample of children across a wider age range to assess whether the associations between ANS acuity, inhibitory control, and math ability operate consistently across development.

## Experiment 2

### Method

#### Participants

A sample of 169 children (4 years 9 months, SD = 8.9 months, 82 females) who took part in a larger longitudinal study on the relation between the ANS and math ability contributed data to the present experiment. Data from other aspects of this study have been reported elsewhere (Libertus et al., 2011, 2013). Testing took place over two sessions. Four children completed only the first session because they were unable to return for the second. Parents of all children tested provided informed written consent prior to their child’s participation. All children received a small gift (e.g., pencil, stickers, stuffed animal, or book) to thank them for their participation.

#### Procedure

Three experienced experimenters conducted all testing sessions, which occurred either in the laboratory or in a quiet room at the children’s preschools. All procedures were approved by Johns Hopkins University’s Institutional Review Board. Testing was divided into two sessions with an average delay of 13.01 days (SD = 12.71 days; range = 0–68 days) between testing sessions. In a few cases where both testing sessions occurred on the same day, children took a break between the two testing sessions to avoid fatigue. During the first session, children completed the same standardized assessment for math ability as in Experiment 1 (TEMA-3; Ginsburg and Baroody, 2003) and then a computerized assessment for inhibitory control (K-CPT; Conners, 2006). This session lasted about 30–45 min. During the second testing session, children completed a number comparison task to assess their ANS acuity similar to the one used in Experiment 1, followed by a working memory task that will not be analyzed for the purposes of the present paper. This second session lasted about 20 min.

#### Number Comparison Task

All aspects of the task were identical to the number comparison task used in Experiment 1, except for the following differences. All stimuli were presented on a 13-inch Apple MacBook laptop screen for 2000 ms. Two different sounds provided feedback throughout the experiment. A high-pitched tone indicated a correct answer; a low-pitched tone indicated an incorrect answer. Children were familiarized to these sounds on six practice trials during which the experimenter provided additional verbal feedback to ensure that children understood the task and were motivated to participate. Following these practice trials, a total of 60 test trials were presented. The number of dots in each collection (blue and yellow) ranged from 4 to 15. On half of the trials (Correlated), the two arrays were equated for individual dot size, whereas on the other half of the trials (Equated), the cumulative surface area of the blue dots and the yellow dots was equated. The default radius of the dots was 60 pixels and the maximum variability in size between the dots was 35%.

#### Inhibitory Control Task

We administered the K-CPT (Conners, 2006) to measure children’s inhibitory control using a simple computerized task. Children saw images of common objects on a computer screen and were asked to push a button every time they saw a picture other than a ball. Each picture was presented for 500 ms, and in different blocks the inter-stimulus interval (ISI) was either 1.5 or 3 s. There were a total of five blocks, with two sub-blocks of 20 trials each for each ISI. The total testing time was 7.5 min.

### Results

#### Data Analyses and Descriptive Statistics

Data was missing for 1, 9, and 11 children for the TEMA-3, number comparison task, and K-CPT respectively. Unlike in Experiment 1, children were significantly more accurate on the Equated trials than on the Correlated trials, *t*(159) = 6.31, *p* < 0.001. Hence, we conducted all of our analyses both on overall accuracy as well as separately for each of the trial types. Children’s overall accuracy on the number comparison task ranged from 40.35 to 98.28%, with an average of 76.10% (SD = 14.00%). Accuracy on the Correlated trials ranged from 28.57 to 100.00% with an average of 73.25% (SD = 15.94), whereas accuracy on the Equated trials ranged from 37.93 to 100.00% with an average of 79.25% (SD = 14.49). Raw scores on the TEMA-3 were converted to standard math ability scores, which ranged from 75 to 145 with an average score of 109.53 (SD = 14.93). Children’s performance on the inhibition task was quantified as the percentage of commission errors, i.e., the percentage of trials on which a child failed to inhibit a response to the picture of a ball. Scores ranged from 4 to 96 with a mean of 57.04 (SD = 24.73).

#### Relations Between ANS Acuity and Math Ability

To examine the association between children’s numerical estimation skills and their math ability, we first calculated bivariate correlations between standard scores on the TEMA-3 and measures of ANS accuracy. There was a significant positive association between accuracy on all trials of the number comparison task and standard math ability scores, *r*(158) = 0.52, *p* < 0.001. This effect was replicated with children’s accuracy in the Correlated and Equated trials as well, *r*(158) = 0.48, *p* < 0.001, and, *r*(158) = 0.56, *p* < 0.001, respectively.

Children’s accuracy in the number comparison task and math ability scores were significantly correlated with children’s age and with inhibition scores (see Table 1). As such, it is possible that the association between children’s numerical estimation skills and math ability was simply an artifact of relations between these confounding variables. To test this claim, we calculated correlations between accuracy on the number comparison task and math ability scores, controlling for age at the first time of testing and inhibition scores. The correlation between overall accuracy and standard math scores remained significant, *r*(145) = 0.45, *p* < 0.001. Additionally, correlations with accuracy on Correlated and Equated trials were also significant even when controlling for age and inhibition scores, *r*(145) = 0.39, *p* < 0.001, and *r*(145) = 0.42, *p* < 0.001, respectively.

**TABLE 1:** **Correlations between math ability, ANS acuity, age at the first assessment, and inhibitory control in Experiment 2**.

****	****	**ANS acuity**		
	**Math ability**	**All trials**	**Correlated trials**	**Equated trials**
Age	0.23**	0.58***	0.48***	0.56***
Inhibitory control	–0.24**	–0.23**	–0.23**	–0.20*

*p < 0.05; **p < 0.01; ***p < 0.001.

To assess whether these associations were consistent across the wider age range of children included in this study, we then separated our sample into two age groups based on a median split (i.e., those 1152–1725 days, *M* = 1523.40 days or 4 years 2 months, SD = 136.95 days, and those 1726–2463 days, *M* = 1952.04 days or 5 years 4 months, SD = 181.78 days) and calculated the bivariate and partial correlations shown above for both younger and older children. The pattern of results did not differ between these two groups.

Zero-order correlations between ANS acuity, math ability, inhibition, and age for both younger and older children are presented in Table 2. Among younger children, ANS accuracy was correlated with math ability scores. Young children’s inhibition scores were correlated with ANS accuracy as well as with math ability scores. Hence, partial correlations were calculated for the subsample of young children as with the full sample of children. Controlling for inhibition and age at assessment, young children’s ANS accuracy was significantly associated with math ability scores, *r*(71) = 0.41, *p* < 0.001. This association held when examining accuracy on Correlated and Equated trials separately, *r*(71) = 0.31, *p* = 0.01, and *r*(71) = 0.44, *p* < 0.001, respectively.

**TABLE 2:** **Correlations between ANS acuity, age at the first session, TEMA-3 scores, and inhibition in Experiment 2**.

****	**1**	**2**	**3**	**4**	**5**	**6**
1. Total ANS accuracy		0.87***	0.88***	0.43***	0.51***	–0.25*
2. Correlated ANS accuracy	0.91***		0.61***	0.31**	0.41***	–0.23*
3. Equated ANS accuracy	0.86***	0.58***		0.44***	0.53***	–0.21^t^
4. Age	0.31**	0.27*	0.28*		0.32**	–0.12
5. Math ability scores	0.50***	0.49***	0.39**	0.04		–0.23*
6. Inhibition scores	–0.25*	–0.24*	–0.20^t^	–0.12	–0.28*	

Values shown above the diagonal refer to the younger subsample (<4 years 9.5 months; n = 75), whereas values below the diagonal refer to the older subsample (n = 74). *p < 0.05; **p < 0.01; ***p < 0.001; ^t^p < 0.10.

Among older children, math ability scores were significantly associated with ANS accuracy, and age was significantly associated with ANS accuracy. However, age was unrelated to children’s math ability scores and to children’s inhibition scores. Additionally, ANS accuracy and math ability scores were significantly related to scores on the inhibition task with the exception of accuracy on the Equated trials, which were marginally associated with inhibition. Similar to the subsample of younger children, we calculated partial correlations for the subsample of older children. Controlling for age and inhibition scores, we found significant associations between math ability and overall accuracy, *r*(70) = 0.48, *p* < 0.001, accuracy on Correlated trials, *r*(70) = 0.47, *p* < 0.001, and accuracy on Equated trials, *r*(70) = 0.36, *p* = 0.002.

### Discussion

The findings of Experiment 2 suggest that children’s ANS acuity is associated with math ability, independent of the effects of inhibitory control even when both inhibitory control and ANS acuity are assessed via a computerized task and when behavioral inhibition is assessed as opposed to interference control. This effect was seen in both trials in which number and surface area were correlated as well as trials in which overall surface area was equated across trials and, further, when the sample was split by age. Thus these results replicated and extended the findings of Experiment 1, showing that ANS acuity across a variety of trial types and ages is related to math ability, even when controlling for inhibitory control.

## General Discussion

The main focus of this study was to examine how ANS acuity and math abilities are related in childhood and, in particular, whether associations between the ANS and math persisted when controlling for children’s inhibitory control abilities. In Experiment 1, we found a significant positive correlation between children’s overall ANS accuracy and scores on a standardized math assessment. Crucially, this association remained significant when controlling for children’s age and inhibitory control—more specifically their interference control, suggesting that, among 5- and 6-year-olds, math-specific cognitive skills are related to formal math abilities. We investigated this association further in Experiment 2 with a larger sample of children, which included a wider age range, and with a computerized measure of inhibitory control that taps into behavioral inhibition. Consistent with the results of Experiment 1, we observed a significant correlation between ANS acuity and math ability that remained significant when holding children’s inhibitory control and age constant. Because children’s performance on the Correlated and Equated trials in the ANS task differed in Experiment 2, we also examined whether the effects of ANS acuity were specific to certain conditions of the task. Math ability was positively and significantly correlated with performance on both Correlated and Equated trials, even when controlling for children’s age and inhibitory control abilities. This pattern of results was also replicated in older and younger subsamples of children. Thus, the present study offers a wealth of evidence that children’s ANS acuity is uniquely related to math ability, regardless of child age, ANS task conditions, or the specific subdomain of inhibitory control assessed. These findings suggest that domain-specific cognitive abilities, specifically the ability to approximate numbers of rapidly encountered events in the environment, are important for children’s early math skills, even when ruling out the effects of domain-general cognitive abilities such as the ability to inhibit irrelevant information.

These results conflict with past research suggesting that associations between formal math and acuity of the ANS are fully attributable to underlying inhibitory control abilities. For example, Fuhs and McNeil (2013) found that math ability was significantly related to performance on the ANS task only in trials in which the more numerous set of dots had less overall surface area. Further, this association was no longer significant when accounting for inhibitory control (i.e., interference control). This study included a sample of low-income children, and thus the difference in the patterns of findings between their study and our own may be attributable to differences in sample characteristics. Most children in the present study were from middle and upper-middle class households, and so it may be that these genuine links between the ANS and early math abilities that are not as strong in children living in more disadvantaged contexts. Thus future research is necessary to examine these processes across a more heterogeneous sample that includes children from a wider range of socioeconomic backgrounds in order to fully understand how and for whom ANS acuity is uniquely related to children’s developing math skills.

In addition to Fuhs and McNeil (2013), Gilmore et al. (2013) argued that associations between ANS acuity and math abilities can be explained by individual differences in inhibitory control. Reconciling our results with those of Gilmore et al. (2013) is more difficult, given the similarities between the two sets of studies such as sample characteristics, measures of ANS acuity, and inhibitory control tasks. Gilmore et al. (2013) found that children’s ANS acuity was related to math ability only for trials in which overall surface area and number were unrelated. These results were not replicated in the present study, as math ability was related to ANS accuracy across different trial types. Additionally, Gilmore et al. (2013) found that overall ANS acuity (i.e., across trial types) was unrelated to math when controlling for inhibitory control (i.e., interference control). Again, these findings were not replicated by the current study; instead, associations between overall ANS acuity, as well as accuracy on each trial type, and math ability remained significant when holding inhibitory control constant. It is unclear why we observed a unique relation between ANS acuity and math ability and others have not, and so future research to explain these differences in results is warranted. However, given the use of two unique samples and different measures of inhibitory control, the present study offers a methodologically strong argument that the ANS is uniquely related to children’s early math abilities.

Interestingly, differences in the patterns of results emerged between Experiments 1 and 2 in the present study. Most notably, in Experiment 1 we observed no significant differences in accuracy across the three trial types (Correlated, Equated, and Anti-correlated), whereas in Experiment 2, children were significantly more accurate on the Equated trials than the Correlated trials. This discrepancy in results was surprising, as the samples and tasks used across the two studies were very similar to one another. However, children in Experiment 2 were slightly younger than children in Experiment 1, and so the effect of trial type may have been particularly strong for younger children and less so for older children. Additionally, task variations, such as the use of three trial types in Experiment 1 and two trial types in Experiment 2 may have influenced children’s performance. As noted above, participants in Experiment 2 were more accurate on the trials in which overall surface area was equated than on the trials in which all dots were the same size. We would have predicted the opposite pattern of performance; that children would find trials in which the side with more dots also had more surface area to be easier. Although in the present study we were unable to further probe this finding, it may be that children used some sort of strategy, such as understanding that if the dots are smaller there will be more of them, to aid performance on this task. However, in Experiment 1 we did not see differences by trial type, possibly due to the fact that children saw three conditions of trials and so the heuristic that was used in Experiment 2 may not have been successful in Experiment 1, hence the difference in findings. Although this is purely speculative and warrants direct empirical work, children’s strategy use could offer a potential explanation for these findings. Finally, children in Experiment 2 received feedback on each trial and, in general, were more accurate than the children in Experiment 1, and so differences in results may stem from these larger differences in performance overall. One additional difference in results was observed between the two experiments: in Experiment 1, ANS acuity and inhibitory control were not correlated with one another, whereas in Experiment 2, these two variables were significantly correlated. These discrepancies in findings could be attributable to differences in the tasks used, such that the inhibition of a behavioral response in the K-CPT is more related to the types of inhibitory control necessary to perform well on the ANS task than inhibition of a verbal response or interference control. However, it is also possible that the stronger correlation observed in Experiment 2 was due in part to the fact that both ANS acuity and inhibitory control were measured using a computerized task. Finally, another possible explanation could be that in Experiment 2 both ANS acuity and inhibitory control were measured in raw units as opposed to age-adjusted scores, like in the NEPSY, and so the confound with age may have strengthen these correlations.

Despite the strengths of the present study, several limitations should be addressed. First, as described above, the samples in both experiments described here were fairly homogenous in terms of socioeconomic status and racial/ethnic status. Especially given that work with low-income children failed to find unique associations between ANS acuity and math (Fuhs and McNeil, 2013), future research with more diverse, heterogeneous samples is necessary. Additionally, although different assessments of inhibitory control were used in each study, measures of ANS acuity and math ability were virtually identical across the two studies, and so future research should address whether this pattern of results is replicated when utilizing other measures of these key constructs. Relatedly, by utilizing different measures of inhibitory control in the two studies, we were unable to compare performance and may have been tapping into slightly different aspects of inhibitory control, such as interference control or behavioral inhibition (Nigg, 2000). Although several studies suggest that these types of measures address a single dimension of inhibitory control (e.g., Archibald and Kerns, 1999; Miyake et al., 2000; Lehto et al., 2003), there is also evidence that there may be important underlying differences across these types of tasks (Nigg, 2000; Huizinga et al., 2006). Administering both tasks, as well as a task that directly required inhibition of irrelevant perceptual features such as the Flanker task (Eriksen and Eriksen, 1974) would potentially yield more fruitful insights into these processes. Finally, both studies were cross-sectional and purely correlational in nature, and so the present study offers limited evidence for causal associations between the ANS and early math skills. Nonetheless, the results of this study suggest that across separate samples and with two distinct measures of inhibitory control, children’s ANS acuity appears to be uniquely related to math ability, independent of the effects of inhibitory control.

If children’s ANS acuity and math abilities are in fact uniquely related, several lines of research warrant further investigation. First, how are the ANS and math abilities related? While the mechanisms to explain these associations are still unknown, several possibilities have been suggested, such as an increased understanding of the relations between numbers and, in particular, ordinality. Previous research with adults has shown that associations between ANS acuity and mental math abilities are mediated by symbolic number ordering abilities, suggesting that ANS may promote an understanding of ordinality of the number system, which, in turn, promotes mathematical abilities (Lyons and Beilock, 2011). Recent work with children suggests that this understanding of ordinality becomes increasingly important for children’s math skills across development, although whether the ANS supports these early symbolic representations is still unclear (Lyons et al., 2014). Alternatively, enhanced acuity of the ANS may support the mapping of number symbols onto their meanings, which then supports early math learning. For example, several studies have found that children who showed a weaker connection between their non-symbolic and symbolic representations of number, as indicated by larger numerical distance effects when comparing Arabic numerals, had lower math scores, suggesting that the mapping between ANS representations and numerals is important for early math learning (Holloway and Ansari, 2009; Mundy and Gilmore, 2009).

Second, correlational results like the ones presented here do not allow us to draw any conclusions about causality and directionality. A few recent training studies provide direct evidence for a causal relation between the ANS and math ability. Park and Brannon (2013) trained adults on a non-symbolic addition and subtraction task for a total of 10 sessions and subsequently found significant improvements on a symbolic arithmetic test. Similarly, Hyde et al. (2014) found that brief training on a non-symbolic number comparison or addition task improved children’s subsequent performance on a symbolic arithmetic test. Additionally, recent findings show that math education also sharpens people’s ANS acuity. Piazza et al. (2013) found that among the Mundurucu—an Amazonian tribe whose language does not have number words larger than 5—the level of math education was significantly correlated with their ANS acuity. The higher people’s education level and thus the greater their exposure to Portuguese, the more precise were their ANS representations.

Finally, further research is needed to understand from where individual differences in the ANS stem. Factors such as individual cognitive abilities (e.g., Blair and Razza, 2007; Clark et al., 2010), parental influences (e.g., Melhuish et al., 2008), and larger socioeconomic characteristics (e.g., Roberts and Bryant, 2011) appear to promote children’s early math abilities, yet it is still unclear what factors contribute to the development of numerical approximation skills and the individual differences observed therein.

In sum, the development of early math abilities are related to number specific cognitive systems such as the ANS. Although more research is needed to understand why and how these processes operate, these associations appear to be unique to ANS acuity and math ability and cannot be explained by more general cognitive factors such as inhibitory control.

### Conflict of Interest Statement

The authors declare that the research was conducted in the absence of any commercial or financial relationships that could be construed as a potential conflict of interest.
